# Epidemiology of Disappearing *Plasmodium vivax* Malaria: A Case Study in Rural Amazonia

**DOI:** 10.1371/journal.pntd.0003109

**Published:** 2014-08-28

**Authors:** Susana Barbosa, Amanda B. Gozze, Nathália F. Lima, Camilla L. Batista, Melissa da Silva Bastos, Vanessa C. Nicolete, Pablo S. Fontoura, Raquel M. Gonçalves, Susana Ariane S. Viana, Maria José Menezes, Kézia Katiani G. Scopel, Carlos E. Cavasini, Rosely dos Santos Malafronte, Mônica da Silva-Nunes, Joseph M. Vinetz, Márcia C. Castro, Marcelo U. Ferreira

**Affiliations:** 1 Department of Parasitology, Institute of Biomedical Sciences, University of São Paulo, São Paulo, São Paulo, Brazil; 2 Department of Parasitology, Microbiology and Immunology, Institute of Biological Sciences, Federal University of Juiz de Fora, Juiz de Fora, Minas Gerais, Brazil; 3 Department of Dermatologic, Infectious and Parasitic Diseases, Faculty of Medicine of São José do Rio Preto, São José do Rio Preto, São Paulo, Brazil; 4 Institute of Tropical Medicine of São Paulo, University of São Paulo, São Paulo, São Paulo, Brazil; 5 Center of Health Sciences and Sports, Federal University of Acre, Rio Branco, Acre, Brazil; 6 Division of Infectious Diseases, Department of Medicine, University of California San Diego, La Jolla, California, United States of America; 7 Alexander von Humboldt Institute of Tropical Medicine and Faculty of Sciences, Department of Cellular and Molecular Sciences, Laboratory of Research and Development, Universidad Peruana Cayetano Heredia, Lima, Peru; 8 Department of Global Health and Population, Harvard School of Public Health, Boston, Massachusetts, United States of America; Federal University of São Paulo, Brazil

## Abstract

**Background:**

New frontier settlements across the Amazon Basin pose a major challenge for malaria elimination in Brazil. Here we describe the epidemiology of malaria during the early phases of occupation of farming settlements in Remansinho area, Brazilian Amazonia. We examine the relative contribution of low-density and asymptomatic parasitemias to the overall *Plasmodium vivax* burden over a period of declining transmission and discuss potential hurdles for malaria elimination in Remansinho and similar settings.

**Methods:**

Eight community-wide cross-sectional surveys, involving 584 subjects, were carried out in Remansinho over 3 years and complemented by active and passive surveillance of febrile illnesses between the surveys. We used quantitative PCR to detect low-density asexual parasitemias and gametocytemias missed by conventional microscopy. Mixed-effects multiple logistic regression models were used to characterize independent risk factors for *P. vivax* infection and disease.

**Principal Findings/Conclusions:**

*P. vivax* prevalence decreased from 23.8% (March–April 2010) to 3.0% (April–May 2013), with no *P. falciparum* infections diagnosed after March–April 2011. Although migrants from malaria-free areas were at increased risk of malaria, their odds of having *P. vivax* infection and disease decreased by 2–3% with each year of residence in Amazonia. Several findings indicate that low-density and asymptomatic *P. vivax* parasitemias may complicate residual malaria elimination in Remansinho: (a) the proportion of subpatent infections (i.e. missed by microscopy) increased from 43.8% to 73.1% as *P. vivax* transmission declined; (b) most (56.6%) *P. vivax* infections were asymptomatic and 32.8% of them were both subpatent and asymptomatic; (c) asymptomatic parasite carriers accounted for 54.4% of the total *P. vivax* biomass in the host population; (d) over 90% subpatent and asymptomatic *P. vivax* had PCR-detectable gametocytemias; and (e) few (17.0%) asymptomatic and subpatent *P. vivax* infections that were left untreated progressed to clinical disease over 6 weeks of follow-up and became detectable by routine malaria surveillance.

## Author Summary

Despite decades of control efforts, malaria remains a major public health concern in Brazil. A large proportion of the 243,000 cases diagnosed per year originate from areas of recent colonization in the densely forested Amazon Basin. This population-based longitudinal study addresses the epidemiology of malaria during the early stages of colonization of frontier settlements in Remansinho area, rural Amazonia. We documented a major decline in the prevalence of *P. vivax* infection, from 23.8% to 3.0%, between March–April 2010 and April–May 2013. Up to 73.1% of the *P. vivax* infections were missed by microscopy as malaria transmission declined and most (56.6%) of these infections caused no clinical signs or symptoms. Few (17.0%) asymptomatic *P. vivax* infections that were left untreated eventually progressed to clinical disease, becoming detectable by routine malaria surveillance, over 6 weeks of follow-up. Moreover, nearly all *P. vivax* infections that were undetected by microscopy had gametocytes, the parasite's blood stages responsible for malaria transmission to mosquito vectors, detected by molecular methods. These findings indicate that apparently healthy carriers of low-density parasitemias, who are often missed by conventional microscopy, contribute significantly to ongoing *P. vivax* transmission and may further complicate residual malaria elimination in Remansinho and similar endemic settings.

## Introduction

Malaria is one of the major tropical infectious diseases for which decades of intensive control efforts have met with only partial success in Brazil [1]. With nearly 243,000 slide-confirmed infections, this country contributed 52% of all malaria cases reported in the Region of the Americas and the Caribbean in 2012 [2]. Most transmission in Brazil occurs in open mining enclaves, logging camps and farming settlements across the Amazon Basin, a region that currently accounts for 99.9% of the country-wide malaria burden [3].

Since the early 1970s, official and informal colonization projects in densely forested areas of Amazonia have attracted migrant farmers from the malaria-free South and Southeast regions, originating a number of new frontier agricultural settlements [4], [5]. Initial land clearing for slash-and-burn agriculture and extensive logging can induce major changes in vector biology, by creating or expanding mosquito breeding habitats, as well as in vector species composition, with a marked increase in the abundance of the highly competent local malaria vector *Anopheles darlingi* [6]–[10]. Not surprisingly, recent frontier settlements, where ongoing deforestation and the immigration of non-immune pioneers favor transmission, constitute malaria hotspots until these communities become more stable and endemicity declines [6].

*Plasmodium falciparum* and *P. vivax* infections are widespread across Amazonia, with rare and focal *P. malariae* transmission [11]–[14]. A clear change has been recently observed in the relative proportion of the two main species. Similar proportions of slide-confirmed infections were due to *P. falciparum* and *P. vivax* until 1990, but transmission of the latter species maintained an upward trend while that of *P. falciparum* declined steadily throughout the next decade [15]. *Plasmodium vivax* now accounts for 85% of the malaria burden in Brazil [2]. These trends may be explained by factors such as the presence of dormant liver stages (hypnozoites) and the early circulation of sexual stages (gametocytes) in peripheral blood, which may render *P. vivax* less responsive than *P. falciparum* to available control strategies based on early diagnosis and treatment of blood-stage infections [16], [17].

Here we describe the epidemiology of malaria and associated risk factors during the early phases of occupation of frontier agricultural settlements in the Amazon Basin of Brazil. We observed a major decline in *P. vivax* prevalence, with vanishing *P. falciparum* transmission, over 3 years of malaria surveillance. Risk of both infection and *P. vivax*-related disease decreased with increasing cumulative exposure to malaria, consistent with anti-parasite and anti-disease immunity being acquired by this population. We discuss the challenges of controlling and eliminating malaria, especially that caused by the resilient parasite *P. vivax*, in low-endemicity areas where most infections are asymptomatic and parasite densities are often below the detection threshold of conventional microscopy.

## Methods

### Study area

**Figure 1:**
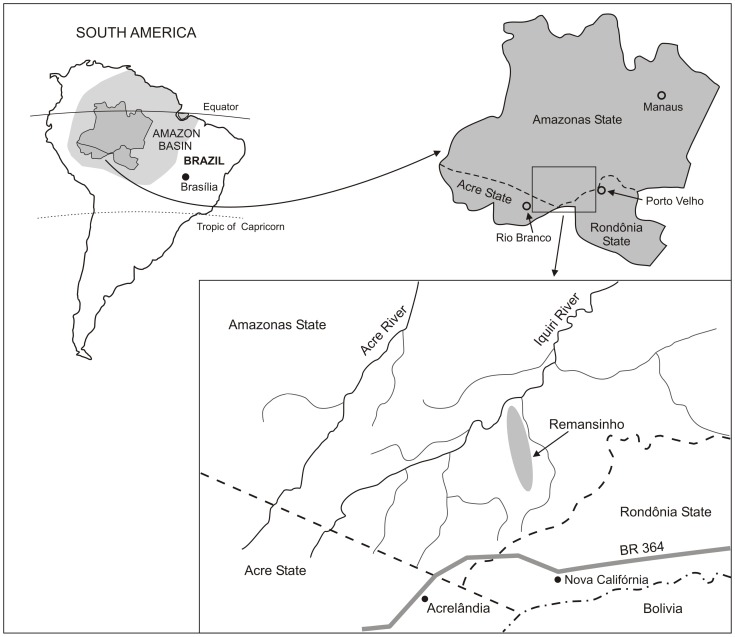
Location of the field site, Remansinho, southern Amazonas State, Brazilian Amazonia. The map also shows the village of Nova Califórnia (western Rondônia State), the nearest town, Acrelândia (eastern Acre State), where our field laboratory is situated, and the BR 364 interstate highway, which connects Acre, Rondônia and southern Amazonas to the rest of the country.

Once a sparsely populated rubber tapper settlement (*seringal*) situated in southern Amazonas state, northwestern Brazil, Remansinho (average population, 260) now comprises five farming settlements (Figure 1). The main settlement is situated along the final 40 km of the Ramal do Remansinho, a 60 km-long unpaved road originating from the BR-364 interstate highway, while the other four are situated along secondary roads (known as Ramal da Linha 1, Ramal da Castanheira, Ramal dos Seringueiros, and Ramal dos Goianos) originating from this main unpaved road (Figure S1). The farming settlements along Ramal da Linha 1 and Ramal da Castanheira were opened in the late 1990s, whereas the colonization of the other areas started only in 2007. Most houses have complete or incomplete wooden walls and thatched roofs; just a few of them have brick walls and are covered with asbestos, cement or zinc shingles. With a typical equatorial humid climate (annual average temperature, 26.4°C), Remansinho receives most rainfall between November and March (annual average, 2,318 mm), but malaria transmission occurs year-round. The main local malaria vector is the highly anthropophilic and exophilic *An. darlingi* [18]. Most families currently living in Remansinho have resettled from other areas within Amazonia, and are now involved in subsistence agriculture and logging. There is a single government-run health post in Remansinho, which provides free malaria diagnosis and treatment, but a small proportion of locally acquired infections are diagnosed and treated in the nearest village (Nova Califórnia; population, 2,600), situated along the BR-364 highway, about 60 km south of Remansinho (Figure 1). There is no electricity or piped water supply in the area.

### Study design and population

A population-based prospective cohort study was initiated in March 2010 to estimate the prevalence and incidence of malaria parasite carriage in Remansinho, by combining microscopy and molecular diagnosis, and to characterize risk factors for malaria infection and clinical disease in the local population. This ongoing study comprises periodic cross-sectional malaria prevalence surveys of the entire population, every four months between March 2010 and March 2011 and every six months thereafter, complemented with clinical and laboratory surveillance of febrile illness episodes between the cross-sectional surveys.

Here we analyze data collected from March 2010 to May 2013. During this period, we enrolled 584 participants belonging to 205 households. Dwellings were geo-localized using a hand-held 12-chanel global positioning system (GPS) receiver (eTrex Personal Navigator, Garmin, Olathe, KS), with a positional accuracy within 15 m. Nearly all (98.8%) study subjects were recruited during house-to-house visits in Ramal do Remansinho (376 or 65.2%), Ramal da Castanheira (85 or 14.7%), Ramal da Linha 1 (57 or 9.9%), Ramal dos Goianos (32 or 5.5%), and Ramal dos Seringueiros (27 or 4.7%); only 7 (1.2%) subjects, who were enrolled at the local health post, had their settlement of origin undetermined.

### Demographic and parasite prevalence surveys

Each cross-sectional survey comprised a population census and the entire population found during the census was considered eligible to participate in the study. During the first (March–May 2010), 165 inhabitants identified during the census in Ramal do Remansinho and Ramal dos Goianos were invited to participate. Subsequent surveys, which also included subjects living in the other three settlements, were carried out between May and July 2010 (survey 2), October and November 2010 (survey 3), March and April 2011 (survey 4), October and November 2011 (survey 5), April and May 2012 (survey 6), October and November 2012 (survey 7), and April and May 2013 (survey 8). Most surveys were carried out either at the beginning or the end of the rainy season, except for survey 2, which took place during the dry season. Total numbers of subjects present in the study area during each survey are given in Table 1.

**Table 1:** Number of malarial infections diagnosed by conventional microscopy (CM) and quantitative real-time PCR (qPCR), according to the presence or absence of malaria-related symptoms, during 8 consecutive cross-sectional surveys in the population of Remansinho, Brazil (2010–13).

		Cross-sectional survey															
		1		2		3		4		5		6		7		8	
Symptoms	Species	CM	qPCR	CM	qPCR	CM	qPCR	CM	qPCR	CM	qPCR	CM	qPCR	CM	qPCR	CM	qPCR
Yes	*P. falciparum*	0	2	0	1	0	3	0	1	0	0	0	0	0	0	0	0
	*P. vivax*	10	20	5	11	3	5	1	2	9	12	1	2	1	3	1	1
	Mixed	0	1	0	0	0	1	0	0	0	0	0	0	0	0	0	0
	No. tested	63	62	54	52	45	42	34	31	40	38	22	21	65	65	46	43
No	*P. falciparum*	1	6	1	10	0	14	0	1	0	0	0	0	0	0	0	0
	*P. vivax*	10	16	10	17	8	14	2	10	4	10	0	3	1	1	0	4
	Mixed	0	1	0	1	0	6	0	0	0	0	0	0	0	0	0	0
	No. tested	96	89	145	135	166	156	182	168	164	159	156	156	134	134	129	126
Total	*P. falciparum*	1	8	1	11	0	17	0	2	0	0	0	0	0	0	0	0
	*P. vivax*	20	36	15	28	11	19	3	12	13	22	1	5	2	4	1	5
	Mixed	0	2	0	1	0	7	0	0	0	0	0	0	0	0	0	0
	No. tested	159	150	199	187	211	198	216	199	204	197	178	177	199	199	175	169
Total population (% tested)		165 (96.3)		276 (72.1)		270 (78.1)		276 (78.3)		290 (70.3)		238 (74.8)		257 (77.4)		221 (79.2)	
Prevalence %	*P. falciparum*	0.6	5.3	0.5	5.9	0.0	8.6	0.0	1.0	0.0	0.0	0.0	0.0	0.0	0.0	0.0	0.0
	*P. vivax*	12.6	24	7.5	15.0	5.2	9.6	1.4	6.0	6.4	11.2	0.6	2.8	1.0	2.0	0.6	3.0
	Mixed	0.0	1.3	0.0	0.5	0.0	3.5	0.0	0.0	0.0	0.0	0.0	0.0	0.0	0.0	0.0	0.0

Dates of cross-sectional surveys were: survey 1 1, March–May, 2010; survey 2, May–July, 2010; survey 3, October–November, 2010; survey 4, March–April, 2011; survey 5, October–November, 2011; survey 6, April–May, 2012; survey 7, October–November, 2012; survey 8, April–May, 2013. Polyethylene bed-nets treated with 2% permethrin (Olyset Net) were distributed to the entire study population in August, 2012.

A baseline questionnaire was applied to all study participants in March–May, 2010, to collect demographic, health, behavioral and socioeconomic data. Cumulative exposure to malaria was estimated using the duration of residence in Amazonia as a proxy. We used a structured questionnaire [19] to determine the presence and intensity of 13 malaria-related signs and symptoms (fever, chills, sweating, headache, myalgia, arthralgia, abdominal pain, nausea, vomiting, dizziness, cough, dyspnea, and diarrhea) up to seven days prior to the interview. Information on selected household assets, access to utilities, infrastructure, and housing characteristics was used to derive a wealth index, from which socioeconomic status was estimated. We combined discrete (i. e., yes or no) ownership information (for power generator, chainsaw, radio, sofa set, shotgun, bicycle, car, motorcycle, and well) and continuous data (i. e., total number of items, for beds, rooms and bedrooms present in the household, and number of pigs, cattle, chickens, ducks, and horses owned). Principal component analysis, carried out using statistical software STATA 12.1, was used to weight each variable [20]. The first principal component explained 18% of the variability and gave the greatest weights to ownership of beds, number of rooms, number of bedrooms, sofa set, and chickens. Lowest weights were given to ownership of horses, ducks or cattle. The scores were summed to give a wealth index for each household. Wealth indices were then used to stratify households into quartiles in increasing order (first quartile, 25% poorest). A shorter version of the baseline questionnaire was used in all subsequent cross-sectional surveys to update demographic and clinical data.

All inhabitants in the study area aged more than 3 months were invited to contribute either venous (5-ml) or finger-prick blood samples for malaria diagnosis, irrespective of any clinical symptoms, Duffy blood group genotyping, and other laboratory assays, such as hemoglobin measurements and ABO and Rh blood group typing. The participation rates ranged between 96.3% in survey 1 (159 of 165 inhabitants) and 70.3% in survey 5 (204 of 290) (Table 1). Nearly all study participants provided venous blood samples in all but one survey; the exception was survey 3, during which finger-prick capillary blood was preferentially collected from all participants for logistic reasons. Reasons for not providing blood samples included temporary absence from the study area, age below 3 months, inability to perform venous puncture, and refusal to participate. Given the high mobility of the study population, only 21 subjects (3.6% of the study population) contributed blood samples in all cross-sectional surveys; 529 subjects (90.6%) participated in two or more surveys. All study participants, either symptomatic or not, who provided either venous or finger-prick blood samples during cross-sectional surveys and had malaria diagnosis confirmed by onsite microscopy were treated according to the malaria therapy guidelines published by the Ministry of Health of Brazil in 2010 [21]. Briefly, *P. vivax* infections were treated with chloroquine (total dose, 25 mg of base/kg over 3 days) and primaquine (0.5 mg of base/kg/day for 7 days), while *P. falciparum* infections were treated with a fixed-dose combination of artemether (2–4 mg/kg/day) and lumefantrine (12–24 mg/kg/day) for 3 days. Infections that were missed by onsite microscopy but later confirmed by polymerase chain reaction (PCR) were left untreated because the results of molecular diagnosis were not available at the time of the cross-sectional surveys.

### Malaria surveillance between cross-sectional surveys

To quantify clinical malaria episodes diagnosed between the cross-sectional surveys, we examined all records of slide-confirmed infections diagnosed between March 2010 and November 2013 at the government-run health posts in Remansinho and in the nearest village, Nova Califórnia. Local malaria control personnel performed both active and passive detection of febrile cases during the study period. Blood samples were collected and examined for malaria parasites whenever febrile subjects visited the health posts in Remansinho or Nova Califórnia or were found during monthly house-to-house visits carried out by field health workers in Remansinho. This strategy is assumed to detect virtually all clinical malaria episodes in cohort subjects between the cross-sectional surveys, since there are no other public or private facilities providing laboratory diagnosis of malaria in the area. Microscopic diagnosis is required to obtain antimalarial drugs in Brazil, which are distributed free of charge by the Ministry of Health and cannot be purchased in local drugstores.

### Laboratory methods

Laboratory diagnosis of malaria was based on microscopic examination of thick smears and PCR. A total of 1,541 thick blood smears were stained with Giemsa in our field laboratory in Acrelândia (120 km southwest of Remansinho). At least 100 fields were examined for malaria parasites, under 1000× magnification, by two experienced microscopists, before a slide was declared negative. We additionally used quantitative real-time PCR (qPCR) that target the 18S rRNA genes [22] to detect and quantify *P. vivax* and *P. falciparum* blood stages in 1,476 clinical samples (Methods S1). Because microscopy is poorly sensitive for detecting circulating gametocytes [23], we used a quantitative reverse transcriptase PCR (qRT-PCR) that targets *pvs25* gene transcripts [24], [25] to detect and quantify mature gametocytes in 55 laboratory-confirmed *P. vivax* infections diagnosed during cross-sectional surveys 4, 5, and 6 (Methods S1).

Since co-infection with multiple parasite clones has been suggested to either increase or reduce the risk of clinical falciparum malaria, we sought to determine whether the presence of multiple-clone *P. vivax* infections was associated with malaria-related morbidity. To this end, we amplified two highly polymorphic single-copy markers, *msp1*F1 (a variable domain of the *merozoite surface protein-1* gene) [26] and *MS16* (a *P. vivax*-specific microsatellite DNA marker with degenerate trinucleotide repeats) [27], using the nested PCR protocols of Koepfli and colleagues [28]. DNA samples from 85 qPCR-confirmed *P. vivax* infections (all of them isolated from venous blood samples) were tested for the presence of multiple clones; 47 were from asymptomatic and 38 from symptomatic parasite carriers. PCR products were analyzed by capillary electrophoresis on an automated DNA sequencer ABI 3500 (Applied Biosystems), and their lengths (in bp) and relative abundance (peak heights in electropherograms) were determined using the commercially available GeneMapper 4.1 (Applied Biosystems) software. The minimal detectable peak height was set to 200 arbitrary fluorescence units. We scored two alleles at a locus when the minor peak was >33% the height of the predominant peak. *Plasmodium vivax* infections were considered to contain multiple clones if one or both loci showed more than one allele.

Since Duffy blood group polymorphisms modulate the ability of *P. vivax* merozoites to invade human red blood cells (reviewed by [29]), we used TaqMan assays (Applied Biosystems) to genotype two major Duffy polymorphisms: the T-33C substitution in the red blood cell-specific GATA1 transcription factor binding motif (rs2814778), which suppresses Duffy expression on the erythrocyte surface (Fy phenotype, associated with FY*B^ES^ allele homozygozity), and the G125A polymorphism (rs12075), which defines the FY*B (wild-type) and FY*A (mutated) alleles associated with the Fyb and Fya phenotypes, respectively. The primers and probes (labelled with VIC and FAM) were designed and synthesized by Applied Biosystems (assay ID, C__15769614_10 and C__2493442_10) [30]. We used a Step One Plus Real-Time PCR System (Applied Biosystems) for genotyping, with a template denaturation step at 95°C for 10 min, followed by 40 cycles of 15 sec at 95°C and 1 min at 60°C, with a final step at 60°C for 30 sec. DNA samples from 487 study participants were genotyped.

### Clinical definitions

A laboratory-confirmed malarial infection was defined as any episode of parasitemia detected by thick-smear microscopy, qPCR, or both. Subpatent or submicroscopic infections were defined as infections confirmed by qPCR but missed by microscopy. We defined clinical malaria as a laboratory-confirmed infection, irrespective of the parasite density, diagnosed in a subject reporting one or more of the 13 signs and symptoms investigated, at or up to seven days before the interview. No attempt was made to calculate pyrogenic thresholds of parasitemias in our heterogeneous group of study participants. Subjects with laboratory-confirmed infection, irrespective of the parasite density, who reported no signs or symptoms at or up to seven days prior to the interview, were classified as asymptomatic carriers of malaria parasites.

### Statistical analysis

A database was created with SPSS 17.0 software (SPSS Inc., Chicago, IL) and all subsequent analyses were performed with R statistical software [31]. For the purposes of explanatory data analysis, proportions were compared using standard χ^2^, Mantel-Haenzel χ^2^ for stratified data, or χ^2^ tests for linear trends. Correlations between parasite densities, which had an overdispersed distribution in the population, and other continuous variables were evaluated using the nonparametric Spearman correlation. Median parasitemias were compared with the nonparametric Mann-Whitney *U* test. Statistical significance was defined at the 5% level and 95% confidence intervals (CI) were estimated whenever appropriate.

Separate regression models were built to describe independent associations between potential risk factors and two outcomes: (a) *P. vivax* infection and (b) clinical (i. e., symptomatic) vivax malaria. Due to the small number of *P. falciparum* infections detected in the community no attempt was made to characterize risk factors for infection with this species. Dependent variables were assumed to follow a binomial distribution with a logit link function, being fitted with a logistic regression. We considered the nested structure of the data, intrinsic to the study design, when building regression models; we have repeated observations (up to 8 observations over 3 years of study; grouping variable, “survey”) nested within subjects (grouping variable, “individual”) who are clustered within households (grouping variable, “household”). This clustered sampling scheme introduces dependency among observations that can affect model parameter estimates. Consequently, we used mixed-effects regression models that include the grouping variables as random factors to handle nested observations.

Our modeling strategy further considered the hierarchical levels of independent variables (Methods S1). The effects of distal determinants, such as demographic, social and environmental factors, on malaria risk are often not direct, but mediated by more proximate determinants, such as occupational and behavioral factors [32]. Variables within each level of determination were introduced in the model in a stepwise approach, and only those that were associated with the outcome at a significance level of at least 20% were retained. Most subjects with missing observations were excluded from the final model, except those with missing values for the following four variables: Duffy genotype, wealth index, whether bedroom windows were left open at night, and main occupation. These were maintained in the model by creating a new missing-value category. All models were adjusted for the timing of the survey (months elapsed since the beginning of the study in March 2010). Three variables in the model were time-dependent: age, years of residence in Amazonia, and timing of the survey. The final models comprised 1,242 observations from 442 individuals grouped into 159 households (outcome: *P. vivax* infection), and 1,237 observations from 438 individuals grouped into 158 households (outcome: vivax malaria).

Alternative logistic models, which excluded Duffy-negative subjects (88 observations from 31 subjects), examined the association between Duffy-positive genotypes (FY*AFY*B^ES^, FY*AFY*A, FY*AFY*B, FY*BFY*B^E^, and FY*BFY*B) and risk of *P. vivax* infection and vivax malaria. To account for the hypothesis that age at the beginning of exposure to malaria affects the rate at which antimalarial immunity is acquired by migrants [33], we further tested for an interaction between age and years of residence in Amazonia. In addition, we fitted mixed-effects Poisson regression models to the data, but the random-effects variances associated with the estimates were substantially higher than those obtained with the logistic models described above. As a consequence, here we only present results derived from the logistic regression analysis.

In addition, we used a mixed-effects Cox proportional hazards model [34] to compare the risk of slide-positive vivax malaria between the surveys in two sub-cohorts of asymptomatic subjects: (a) carriers of subpatent *P. vivax* infections at baseline that were left untreated and (b) control subjects who were parasite-negative at baseline by both microscopy and qPCR. Subjects who were symptomatic but parasite-negative at baseline were excluded from the uninfected sub-cohort because they might harbor ongoing low-grade infections, causing malaria-related symptoms, which were missed by our laboratory methods. At each survey, eligible study participants were assigned to either sub-cohort and followed up until the next survey at which their clinical and infection status was reassessed. Time at risk was defined as either the interval between two consecutive surveys in which the subjects participated (the first survey in the pair was defined as the baseline survey) or the interval between the baseline survey and the date when subjects left the study, whatever came first. Analysis was adjusted for subjects' age (stratified as <15 years and ≥15 years), Duffy blood group negativity, and years of residence in Amazonia. The clustering of repeated observations within individuals was modeled as a random effect [34].

As required for all observational studies published by *PLoS Neglected Tropical Diseases*, this article includes the STROBE (STrengthening the Reporting of OBservational studies in Epidemiology) checklist to document its compliance with STROBE guidelines (Checklist S1).

### Ethics statement

Study protocols were approved in early 2010 by the Institutional Review Board of the University Hospital of the University of São Paulo (1025/10) and by the National Human Research Ethics Committee of the Ministry of Health of Brazil (551/2010). The ethical clearance has been renewed annually by the Institutional Review Board of the University Hospital of the University of São Paulo. Written informed consent was obtained from all study participants or their parents/guardians.

## Results

### Subject characteristics and prevalence of malaria infection

Of 584 people living in Remansinho who participated in at least one cross-sectional survey, 333 (57.0%) were male and 251 (43.0%) were female, with a median age of 23.0 years. Nearly all (94.3%) adult subjects aged more than 18 years were migrants, 42.2% of them originating from malaria-free areas outside Amazonia. Only 31 subjects (6.4%) were homozygous FY*B^ES^ carriers, with the *P. vivax*-refractory Duffy-negative (Fy) phenotype; 127 (26.1%) had the Fya phenotype (70 FY*AFY*B^ES^ heterozygotes and 57 FY*A FY*A homozygotes), 142 (29.2%) had the FyaFyb phenotype (FY*A FY*B heterozygotes), and 187 (38.4%) had the Fyb phenotype (91 FY*BFY*B^ES^ heterozygotes and 96 FY*B FY*B homozygotes).

Polyethylene bed-nets treated with 2% permethrin (Olyset Net, Sumitomo Chemical, London, United Kingdom) were distributed, free of charge, to the entire study population in August 2012, as a component of malaria control activities in Brazilian Amazonia. In October–November 2012 (survey 7), 74.4% of the study participants reported having slept the previous night under an Olyset net; the corresponding figure for April–May 2013 (survey 8) was 84.5%. No other insecticide-treated bed nets were available in the community.

A total of 1,541 blood samples were examined for malaria parasites by microscopy, qPCR, or both. Of these, 141 (9.1%) were positive (by one or both methods) for *P. vivax*, 40 (2.6%) for *P. falciparum* and 10 (0.6%) for both species. Over the entire study period, 191 (12.4%) samples examined tested positive for malaria parasites; 10 *P. vivax* and 2 *P. falciparum* infections were only diagnosed by microscopy, since DNA samples were not available for qPCR or qPCR yielded negative results. In addition, 61.8% of all infections diagnosed by qPCR, regardless of the infecting species, and 49.6% of the qPCR-confirmed single-species *P. vivax* infections, were missed by conventional microscopy and thus defined as subpatent. The last *P. falciparum* infections in Remansinho were diagnosed (by qPCR only) in March–April 2011.

These figures, however, changed over time. The numbers of malaria infections, either symptomatic or not, diagnosed by conventional microscopy and qPCR in each cross-sectional survey are shown in Table 1. The proportions of qPCR-confirmed single-species *P. vivax* infections that were subpatent varied widely across surveys, ranging from 73.1% in the surveys with the lowest *P. vivax* prevalence rates (surveys 4, 6, 7, and 8 combined; 26 qPCR-confirmed infections) to 43.8% in those with the highest prevalence rates (surveys 1, 2, 3, and 5 combined; 105 qPCR-confirmed infections; Yates' corrected χ^2^ = 6.02, 1 degree of freedom [df], *P* = 0.014). The numbers of *P. falciparum* and mixed-species infections were too small for a similar comparison. Microscopy thus had a better diagnostic performance for vivax malaria when overall parasite prevalence rates were higher, consistent with a recent meta-analysis of *P. falciparum* data showing lower proportions of submicroscopic infections in areas with greater malaria transmission [35].

### Malaria-related symptoms, parasite density, and gametocytes

Overall, 17.1% of the study subjects (ranging between 12.3% in survey 6 and 39.6% in survey 1) interviewed during the cross-sectional surveys reported one or more malaria-related signs and symptoms up to seven days prior to the interview (Table 1). However, reported clinical signs and symptoms were neither sensitive nor specific for malaria diagnosis. On the one hand, almost two thirds (64.5%) of all qPCR-confirmed malaria infections by any species, and 56.6% of those due to *P. vivax*, were asymptomatic; on the other hand, only 26.7% of subjects reporting symptoms had a malaria infection (by any species) confirmed by microscopy, qPCR, or both. All carriers of mixed-species infections (all of them confirmed by qPCR but missed by microscopy) were asymptomatic (Table 1).

Most *P. vivax-*infected subjects harbored few parasites, with densities estimated by qPCR on 129 samples ranging between 2.1 and 38,390 parasites/µL (median, 49.1 parasites/µL; interquartile range, 10.0–483.1 parasites/µL; data were missing for 2 qPCR-confirmed infections). We found no evidence for decreasing *P. vivax* densities with increasing cumulative exposure to malaria in this population. In fact, individual *P. vivax* parasitemias did not show a negative correlation with the subjects' length of residence in Amazonia, a proxy of cumulative exposure to malaria (Spearman correlation coefficient *r_s_* = −0.046, *P* = 0.600), or with their age (*r_s_* = −0.068, *P* = 0.427). We next tested whether differences in the diagnostic sensitivity of conventional microscopy across cross-sectional surveys might be explained by higher average parasite densities found at times of increased malaria transmission [35]. Parasitemias appeared slightly higher in qPCR-positive samples (*P. vivax* only) obtained during surveys 1,2,3 and 5 (high prevalence), with a median of 55.7 parasites/µL (interquartile range, 10.4–597.6 parasites/µL; n = 103), than in those obtained during surveys 4,6,7, and 8 (low prevalence), with a median of 19.8 parasites/µL (interquartile range, 5.8–65.4 parasites/µL; n = 26), although the difference did not reach statistical significance (Mann-Whitney U test, *P* = 0.057).

**Figure 2:**
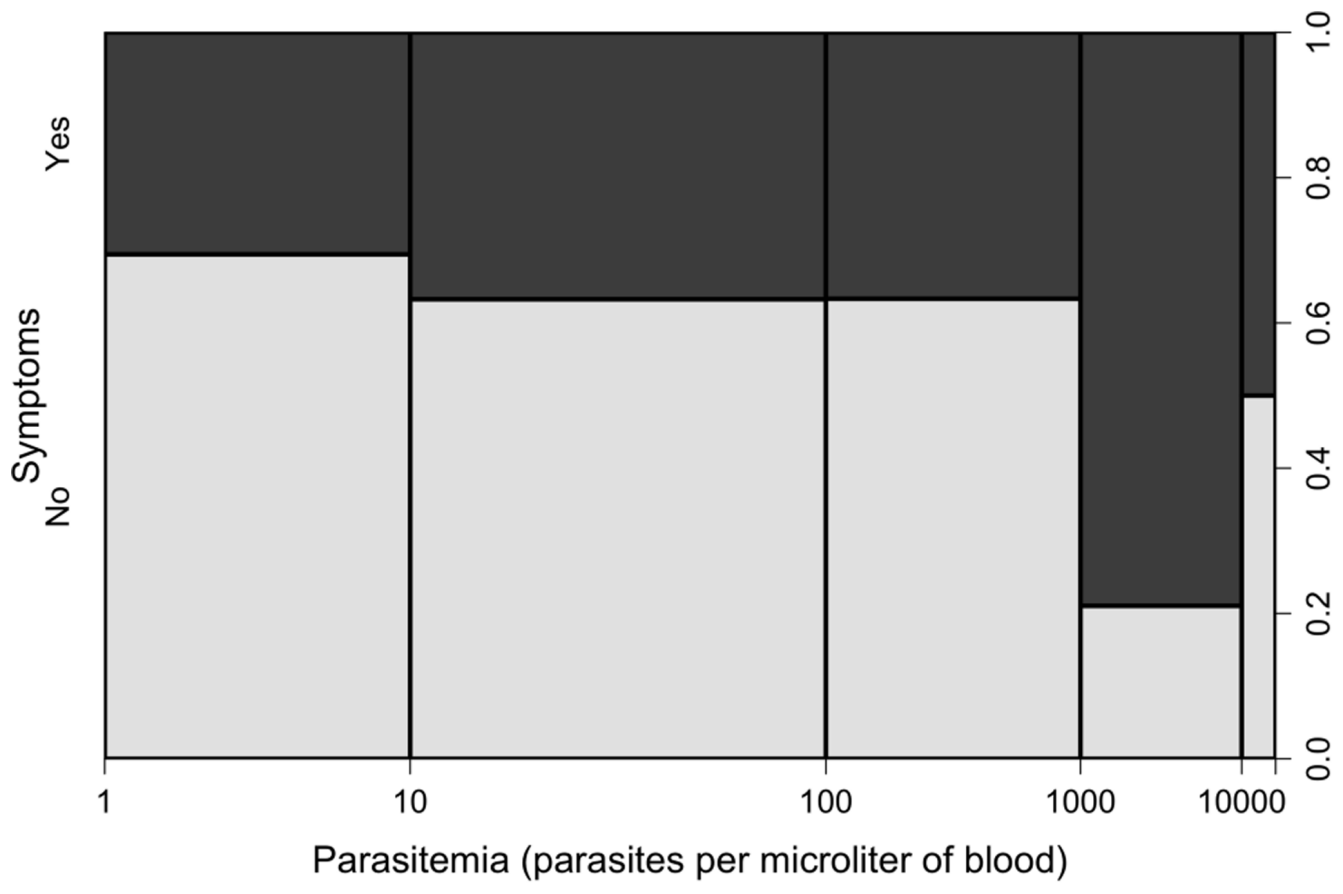
Proportion of *P.vivax* infections diagnosed during 8 consecutive cross-sectional surveys in Remansinho, Brazil, that were symptomatic (black bar segments) and asymptomatic (white bar segments) according to parasite density estimated by quantitative PCR. The bar widths are proportional to the number of cases within each parasite density class. A total of 129 *P. vivax* infections were classified according to the presence of symptoms and parasite density.

**Figure 3:**
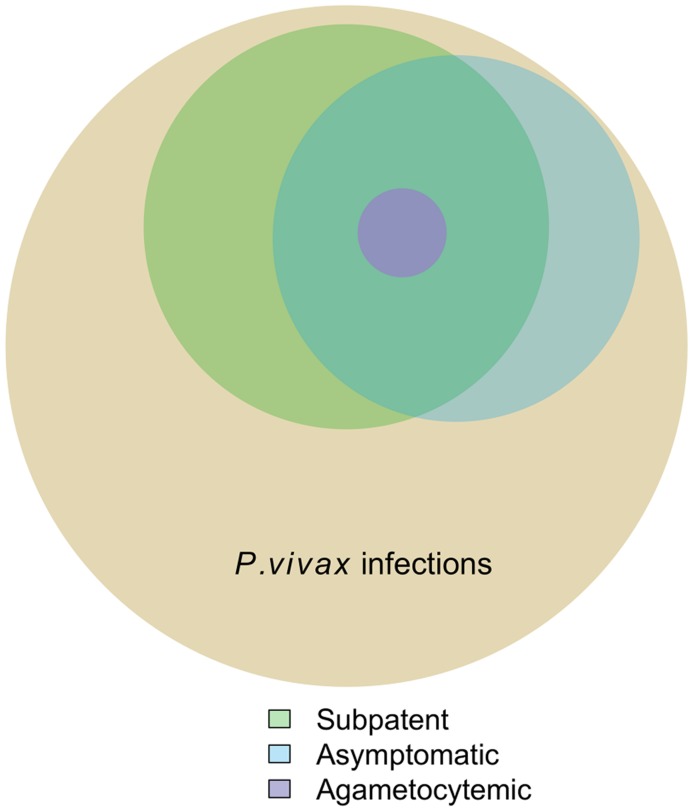
Venn diagram showing the proportion of *P. vivax* infections diagnosed by quantitative PCR during 8 consecutive cross-sectional surveys in Remansinho, Brazil, that were asymptomatic, subpatent (i.e., missed by conventional microscopy) and agametocytemic. The latter group comprises infections with no *pvs25* gene transcripts detected by quantitative reverse transcriptase PCR; note that all agametocytemic infections were both asymptomatic and subpatent.

The proportion of symptomatic *P. vivax* infections correlated positively with increasing parasite density (χ^2^ for trend = 7.99, 1 df, *P*<0.005). Only 30.6% of the subjects carrying less than 10 parasites/µL, but 73.9% of those carrying more than 1,000 parasites/µL, reported one or more malaria-related symptoms (Figure 2). Consistent with previous observations from Amazonia [36], [37], more than half (53.9%) of the asymptomatic infections with this species confirmed by qPCR were missed by conventional microscopy (Table 1). Overall, 32.8% of the 131 single-species, qPCR-confirmed *P. vivax* infections for which complete data were available were both subpatent and asymptomatic (Figure 3). Only one *P. vivax* infection was diagnosed by qPCR, but missed by conventional microscopy, among 88 samples collected from Duffy-negative study participants during the 8 cross-sectional surveys. The only reported symptom during this subpatent *P. vivax* infection in a Duffy-negative subject was a chronic myalgia; parasite density was very low (9.9 parasites/µL of blood).

To estimate the relative contribution of asymptomatic parasite carriage to the total *P. vivax* biomass in the host population, we summed up all individual qPCR-derived *P. vivax* densities and calculated the fraction corresponding to asymptomatic infections. Assuming that, on average, asymptomatic and symptomatic subjects have similar whole blood volumes, we concluded that most (54.4%) *P. vivax* blood stages circulating in Remansinho at the time of the surveys were found in apparently healthy subjects who were unlikely to have their infection diagnosed through case detection strategies targeting only febrile subjects. The number of *P. falciparum* and mixed-species infections was too small for similar analyses.

Next, we examined whether *P. vivax* gametocyte carriage was similarly frequent in symptomatic and asymptomatic infections. We detected *pvs25* gene transcripts, consistent with mature *P. vivax* gametocytes circulating in the bloodstream, in all 32 symptomatic infections, and in 21 of 23 (91.3%) asymptomatic infections from which cryopreserved blood samples were available for RNA extraction. Interestingly, 25 of 27 (92.6%) subjects with subpatent *P. vivax* parasitemia, and all 28 subjects with patent infection, had *pvs25* gene transcripts detected by qRT-PCR. Therefore, qRT-PCR failed to detect *pvs25* transcripts in only 2 (3.6%) of 55 samples tested (Figure 3), both of them collected from asymptomatic carriers of low parasitemias (6.3 and 11.0 parasites/µL). Not surprisingly, the number of *pvs25* transcripts per µL of blood, measured by qRT-PCR, correlated positively with the qPCR-derived overall parasite density (*r_s_* = 0.445, *P*<0.0001).

### Risk factors for *P. vivax* infection and disease

**Figure 4:**
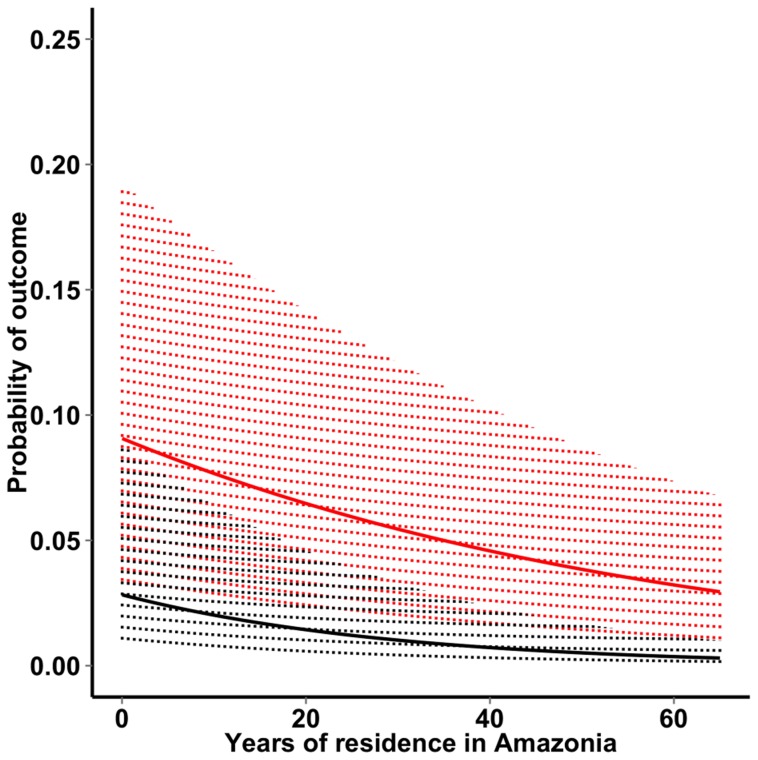
Correlation between length of residence in Amazonia (in years), a proxy of cumulative exposure to malaria, and the probability of having a *P. vivax* infection (continuous red line) and a clinical vivax malaria episode (continuous black line). Lines represent median individual probabilities derived from the final (fully adjusted) mixed-effects logistic regression models; the shaded area surrounding the lines represent interquartile ranges.

The mixed-effects logistic regression model showed that the risk of *P. vivax* infection decreased with increasing cumulative exposure to malaria, consistent with anti-parasite immunity being acquired in this population (Table 2). Each additional year of residence in Amazonia decreased the average odds of being infected by 2% (Figure 4). There was no significant interaction between age and years of residence in Amazonia (*P* = 0.9008), suggesting that the subjects' age when exposure started did not affect, in this migrant population, the rate of decline in *P. vivax* infection risk with increasing time of residence in Amazonia. Calendar time was also a major determinant of infection risk; each month elapsed since March 2010 was associated with a 7% decrease in the odds of being infected (Table 2). Moreover, the grouping variable “survey” accounted for 99.9% of the random effect variance in the mixed-effects model, with minor contribution of individual- and household-level grouping.

**Table 2:** Factors associated with *Plasmodium vivax* infection and clinical vivax malaria, as revealed by multiple logistic regression analysis, in Remansinho, Brazil (2010–13).

	*Plasmodium vivax* infection		Clinical vivax malaria	
	OR^a^ (95% CI^b^)	*P* value	OR^a^ (95% CI^b^)	*P* value
Duffy blood group phenotype				
Positive	1 (reference)		1 (reference)	
Negative	0.08 (0.01–0.60)	0.0142	0.16 (0.02–1.29)	0.0849
Missing information	0.80 (0.32–2.00)	0.6362	0.41 (0.05–3.32)	0.4015
Length of residence in Amazonia (years)	0.98 (0.97–1.00)	0.0238	0.97 (0.94–0.99)	0.0082
Time since beginning of study (months)	0.93 (0.90–0.96)	<0.0001	0.92 (0.87–0.98)	0.0062
Main Occupation				
Housekeeping	1 (reference)		1 (reference)	
Agriculture and forestry	0.83 (0.52–1.32)	0.4325	0.97 (0.48–2.00)	0.9473
Students and minors	0.39 (0.20–0.76)	0.0058	0.26 (0.09–0.74)	0.0125

a OR = adjusted odds ratio.

b CI = confidence interval.

Interestingly, adjusting for more proximate determinants affected the association between age and risk of infection with *P. vivax* in multivariate models. Age under 15 years was a protective factor of borderline significance (partially adjusted OR = 0.616; 95% CI, 0.33–0.95, *P* = 0.057) in the first model, which also adjusted for sex, years of residence in Amazonia, Duffy blood group genotype, months elapsed since the beginning of the study, and wealth index quartiles. However, after adjusting for main occupation, the effect of age on infection risk became non-significant (fully adjusted OR = 1.169; 95% CI, 0.63–2.19, *P* = 0.624). These results indicate that young age *per se* is not protective, but young subjects are less likely to engage in activities such as logging and fishing in the fringes of the rain forest, which are potentially associated with increased risk of infection (Table 2).

Not surprisingly, Duffy-negativity emerged as a protective factor against *P. vivax* infection in this community (Table 2). However, additional logistic regression models including only Duffy-positive subjects showed that, compared to FY*A FY*B heterozygotes, neither FY*A FY*B^ES^ heterozygotes (OR = 0.864; 95% CI, 0.43–1.73) and FY*A FY*A homozygotes (OR = 0.921; 95% CI, 0.48–1.75) were protected against *P. vivax* infection, nor FY*B FY*B^ES^ heterozygotes (OR = 1.226; 95%, 0.69–2.17) and FY*B FY*B homozygotes (OR = 0.588; 95% CI, 0.32–1.09) were at increased risk of infection. These results are consistent with a protective role of FY*B^ES^ heterozygosity, but not of FY*A allele carriage, against *P. vivax* infection in this population.

The risk of clinical *P. vivax* malaria decreased with increasing cumulative exposure to malaria (Table 2); each additional year of residence in Amazonia decreased the odds of having vivax malaria by 3%, again with no significant interaction between age and length of residence in Amazonia (*P* = 0.863). These findings are consistent with similar exposure-dependent rates of acquisition of anti-parasite and anti-disease immunity in this community. Calendar time was the only other major determinant of malaria risk; each month elapsed since the beginning of the study was associated with an 8% decrease in the odds of having clinical vivax malaria (Table 2). Due to the small sample size, Duffy-negativity emerged as a protective factor of borderline significance (OR = 0.16, 95% CI, 0.02–1.29, *P* = 0.084) against clinical vivax malaria.

### Asymptomatic *P. vivax* carriage and subsequent risk of clinical malaria

**Figure 5:**
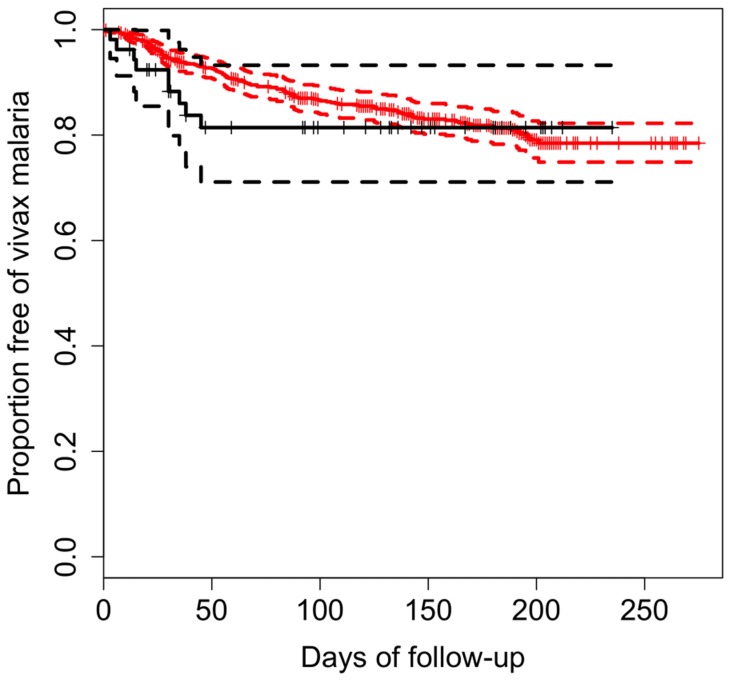
Kaplan-Meier estimates of the proportion of *P. vivax*-infected (continuous black line) and uninfected (continuous red line) asymptomatic subjects who remained free of slide-confirmed clinical vivax malaria over the follow-up period. Dashed lines represent the respective 95% confidence intervals. The small vertical tick-marks indicate the occurrence of a slide positive case of *P.vivax*, corresponding to the right censoring of the individual survival time. A Cox proportional hazards model revealed no significant difference, between the two groups, in overall risk of vivax malaria episodes, after controlling for potential confounders (hazard ratio = 1.07; 95% CI, 0.52–2.22, *P* = 0.840).

In Brazil, malaria is only treated if blood smear microscopy is positive; subpatent malaria parasitemia as determined with qPCR is not accepted as the basis for treatment. Of 53 asymptomatic subpatent *P. vivax* infections diagnosed at baseline, 9 (17.0%) progressed to clinical malaria over the following 6 weeks, being diagnosed by onsite microscopy and treated (Figure 5). During this 6-week period, only 2.5% of the subjects in the uninfected cohort experienced an episode of slide-confirmed vivax malaria, but at the end of the follow-up period similar proportions of subjects in each sub-cohort had experienced vivax malaria episodes confirmed by microscopy (Figure 5). A Cox proportional hazards model revealed no significant difference, between the two sub-cohorts, in overall risk of vivax malaria episodes, after controlling for potential confounders (hazard ratio = 1.07; 95% CI, 0.52–2.22, *P* = 0.840). Most subpatent asymptomatic infections cleared spontaneously (or, at least, became undetectable by qPCR), since only 5 of 44 (11.4%) carriers who remained untreated were again *P.* vivax-positive in the next survey. Therefore, few asymptomatic and subpatent *P. vivax* infections eventually became patent and symptomatic (and therefore detectable by routine malaria surveillance) over the following weeks. We conclude that untreated, low-density, and asymptomatic *P. vivax* parasitemias may persist for several weeks without progressing to clinical disease, and thus constitute a major infectious reservoir for continued transmission in the community.

### Multiple-clone *P. vivax* infections and malaria-related illness

By typing two highly polymorphic markers, we found more than one genetically distinct clone in 25 of 85 (29.4%) *P. vivax* infections analyzed. Although multiple-clone infections were more frequent in symptomatic (13 of 38, 34.1%) than asymptomatic (12 of 47, 25.5%) carriers, this difference did not reach statistical significance (Yates' corrected χ^2^ = 0.762, 1 df, *P* = 0.382). Because average *P. vivax* densities were lower in asymptomatic infections and detecting minority clones may be more difficult in samples with low-level parasitemias, we re-analyzed the data after stratifying parasite densities into quartiles. Again, stratified analysis yielded negative results (Mantel-Haenzel χ^2^ = 0.004, 1 df, *P* = 0.991). Therefore there was no observable association between multiplicity of *P. vivax* infection and the presence of symptoms in this community.

## Discussion

This longitudinal study in newly opened frontier settlements provides further evidence that carriers of low-density parasitemias, who are often missed by conventional microscopy, contribute significantly to ongoing *P. vivax* transmission in rural Amazonia. Results from this and other studies in Amazonia [12]–[14], [36], [37] challenge the often persisting view that subjects in low malaria transmission settings are unlikely to harbor low parasitemias, due to the lack of acquired immunity. To the contrary, average parasite densities decreased, with higher proportions of *P. vivax* infections being missed by microscopy, as malaria prevalence decreased in the community. Interestingly, our findings for *P. vivax* are consistent with a recent meta-analysis of 106 *P. falciparum* prevalence studies worldwide that combined microscopy and molecular methods [35]. Because the risk of *P. vivax* infection (confirmed by microscopy, qPCR, or both) correlated negatively with cumulative exposure to malaria, we suggest that our study population has developed over time some degree of anti-parasite immunity, in line with recent findings from traditional riverine communities in Amazonia [13], [37]. Finally, we show that nearly all subpatent blood-stage *P. vivax* infections comprise mature gametocytes detected by a highly sensitive molecular technique [24]. We thus conclude that subpatent infections constitute a major *P. vivax* reservoir in rural Amazonia and possibly in other low-transmission settings.

Our findings also challenge classical views regarding asymptomatic infections in low-endemicity populations. Prior to the molecular diagnosis era, nearly all laboratory-confirmed episodes of malarial infection, even those with low parasite densities, were thought to elicit clinical disease in pioneer settlements across the Amazon Basin [38]–[40]. More recent surveys, however, demonstrated that subclinical infections are common in agricultural settlements [12], [14] and traditional riverine communities [13], [36], [37], [41], but most of them are missed by microscopy. Interestingly, the high proportion of infections found to be asymptomatic in the present study must be interpreted as a conservative estimate. We may have misclassified some episodes of parasite carriage in subjects reporting any of the 13 symptoms investigated, which may or may not be caused by the current infection, as symptomatic malaria infections, overestimating the proportion of symptomatic infections. Not surprisingly, however, we found very low *P. vivax* densities in most subclinical infections in Remansinho. Conventional microscopy missed 54% of them, suggesting that previous microscopy-based studies failed to detect asymptomatic parasite carriage in rural Amazonians because they missed a large proportion of low-density infections. Mathematical models identified asymptomatic infections as a crucial target for *P. falciparum* malaria eradication efforts in Africa [42], but no similar analyses are available for other endemic areas and other human malaria parasite species [43]. The following findings argue for a major role of asymptomatic infections in maintaining ongoing *P. vivax* transmission in Remansinho: (a) apparently healthy subjects accounted for half of the total *P. vivax* biomass found in the local population, (b) nearly all asymptomatic infections comprised mature gametocytes, and (c) few untreated asymptomatic infections became symptomatic (and thus detectable by routine surveillance) over the next few weeks of follow-up. We were unable to measure the average duration of untreated, asymptomatic infections in our population; there is a recent estimate of 194 days of duration for untreated *P. falciparum* infections in Ghana [44], but no comparable estimate is currently available for *P. vivax*. Specific studies to quantify the transmissibility of subpatent parasitemia to vector mosquitos via direct and membrane feeding assays are ongoing (JMV and colleagues, unpublished data).

Who are at risk of malaria in Remansinho? Migrants from malaria-free areas (54.5% of the adults in the community) constitute a major risk group, with each year of residence in Amazonia decreasing their risk of *P. vivax* infection and clinical vivax malaria by 2–3%. In some Amazonian communities, malaria has been associated with forest-related activities such as logging, fishing and mining, which typically involve young male adults [12], [39], [45], [46]. However, we show that housekeeping and forest-related activities were associated with similar risks for infection and disease in Remansinho. We hypothesize that nearly all adolescents and adults of both genders engage to some extent in farming activities, especially harvesting, in the forest fringes close to their dwellings, although only young males are often involved in logging and land clearing in more densely forested areas. We are currently using high-resolution satellite images to measure the distance between dwellings and forest fringes to further explore the association between proximity to the forest environment and risk of malaria in Remansinho. Interestingly, malaria transmission appears to be relatively homogeneous across all settlements in the area, equally affecting the poorest and least poor people of both sexes, with no differences in risk according to main house characteristics. Whether the vectorial capacity of *An. darlingi* is spatially homogeneous is a key question to be answered by ongoing vector biology studies in this site.

Detection of gametocytes, through *pvs25* gene transcripts, in nearly all qPCR-confirmed *P. vivax* infections tested is somewhat surprising, since recent studies have found much lower proportions of gametocyte-positive infections in Southeast Asia [47], [48] and Papua New Guinea [49]. Since gametocytes comprise only 2% of circulating blood stages [50], microscopists are likely to miss gametocytes in population-based studies where low-density infections are often sampled [23]. Furthermore, we argue that even molecular methods may be poorly sensitive if suboptimal techniques for sample storage and RNA extraction are used under field conditions. For RNA isolation, we cryopreserved venous blood samples at −70°C or in liquid nitrogen a few hours after collection, since our previous attempts to amplify *pvs25* transcripts from RNA isolated from classic FTA microcards (Wathman), QIAcards (Qiagen), 903 protein saver cards (Whatman), and 3MM filter papers (Whatman) impregnated with *P. vivax*-infected blood and kept at ambient temperature had all failed [24]. Storing filter papers impregnated with blood in TRizol reagent (Qiagen) may improve RNA yield, but almost two thirds of the bloodspots from PCR-confirmed *P. vivax* infections tested by Wampfler and colleagues [49] were negative for *pvs25* transcripts by TaqMan assays, despite previous TRizol reagent treatment.

Long-term asymptomatic carriage of *P. falciparum* has been suggested to protect against subsequent malaria-related disease in Africa [51], [52], possibly by reducing the risk of superinfection with more virulent strains. An explanation for this finding is premunition, originally defined by Sergent and Parrot (1935) as the protection against new infections resulting from immune responses to the existing infection [53]. Alternatively, ongoing blood-stage infection might arrest the development of subsequently inoculated sporozoites in the liver. Such an inhibition of superinfection appears to be mediated by the iron regulatory hormone hepcidin, produced in response to blood-stage parasitemia [54]. However, an opposite effect (i.e., increased risk of subsequent disease in asymptomatic *P. falciparum* carriers) has also been described, suggesting that a proportion of asymptomatic infections will eventually reach the host's pyrogenic threshold [55]. Here we found no significant association between asymptomatic carriage of low-density *P. vivax* infection and protection from subsequent malaria morbidity, suggesting that treating individuals with asymptomatic *P. vivax* infections would not render them more vulnerable to clinical malaria over the next few weeks or months.

Although we have identified challenges for malaria control that are not currently addressed by routine surveillance, malaria transmission in Remansinho has declined dramatically over 3 years of surveillance, and *P. falciparum* was found only during the first four surveys. Factors that may have contributed to this decline include drastic environmental changes resulting from logging and land clearing for farming, variation in climate, the widespread use of insecticide-treated bed-nets since August 2012, and the implementation of research activities in the area.

To address the first two hypotheses, we are now analyzing high-resolution satellite images to track environmental changes over time. Consistent with the third hypothesis, two studies have provided evidence that insecticide-treated bed-nets are effective for malaria control in Amazonia. The first was a case-control study in Colombia that showed more than 50% reduction in malaria, relative to no net use, although the advantage of impregnated over non-impregnated nets was not statistically significant [56]. The second study, a randomized trial of lambdacyhalothrin- versus placebo-treated nets in the Amazonas State of Venezuela, showed a protective efficacy of 55% [57]. Whether insecticide-tread bed-nets alone can reduce malaria incidence rates throughout the Amazon Basin remains uncertain, mostly due to the highly variable biting behavior of *An. darlingi* across the region [58], with strong evidence of significant blood-fed and exophilic host-seeking behavior [59]–[61]. In addition, the decline in transmission in Remansinho preceded the distribution of bednets. Finally, the presence of a research team continuously working in the area for over 3 years may affect positively both diagnostic and treatment practices. The external slide revision routinely carried out by our team provides an example of intervention that may have enhanced the diagnostic skills of local microscopists. Moreover, active case detection during 8 consecutive surveys allowed for the early diagnosis and prompt treatment of several slide-positive asymptomatic infections that would have been missed by routine passive surveillance.

Eliminating residual foci when malaria is nearly disappearing, but remains entrenched in a few hotspots, is the next major goal in Remansinho and many other similar endemic settings. Case detection strategies in areas approaching malaria elimination often target only subjects presenting with fever or with a history of recent fever, who are screened for malaria parasites by conventional microscopy or rapid diagnostic tests (RDT) and receive prompt antimalarial treatment if found to be infected [62]. These strategies overlook asymptomatic infections that might be detected by periodic cross-sectional surveys of the entire population at risk [63], as we did in Remansinho. Nevertheless, the cost-effectiveness of mass blood surveys for detecting and treating these residual infections decreases proportionally as malaria transmission declines, since: (a) large populations must be screened to diagnose relatively few asymptomatic carriers, and (b) diagnostic techniques available for large-scale use, such as microscopy and RDT, are not sensitive enough to detect low-grade infections that are typical of residual malaria settings [64]. As an alternative, we are currently testing a reactive case detection strategy that has been tailored for the relapsing parasite *P. vivax* to detect new infections in the neighborhood of malaria cases diagnosed by routine surveillance in frontier settlements similar to Remansinho. Evaluating this and other strategies of active surveillance to cope with asymptomatic infections in residual *P. vivax* foci is a top research priority in the context of current malaria elimination efforts worldwide.

## Supporting Information

Figure S1 Map showing the five human settlements within Remansinho area. Open circles are approximate locations of the households with study subjects.
(TIF)

Figure S2 Conceptual hierarchical framework used to evaluate risk factors for *P. vivax* infection and clinical vivax malaria in mixed effects logistic regression models.
(TIF)

Checklist S1 STROBE (STrengthening the Reporting of OBservational studies in Epidemiology) checklist. As required for all observational studies published by *PLoS Neglected Tropical Diseases*, this paper includes the STROBE checklist to document its compliance with STROBE guidelines.
(DOC)

Methods S1 Detailed description of the molecular methods used to detect malaria parasites and gametocytes, as well as of the hierarchical approach used to model risk factors for *P. vivax* infection and clinical vivax malaria. This supplement includes **Figure S2**.
(DOCX)
