# Supplementary figures and images for: Epidemiology of Disappearing Plasmodium vivax Malaria: A Case Study in Rural Amazonia
Source: PLoS Negl Trop Dis. 2014 Aug 28;8(8):e3109. doi: 10.1371/journal.pntd.0003109 (PMC4148206; doi:10.1371/journal.pntd.0003109)

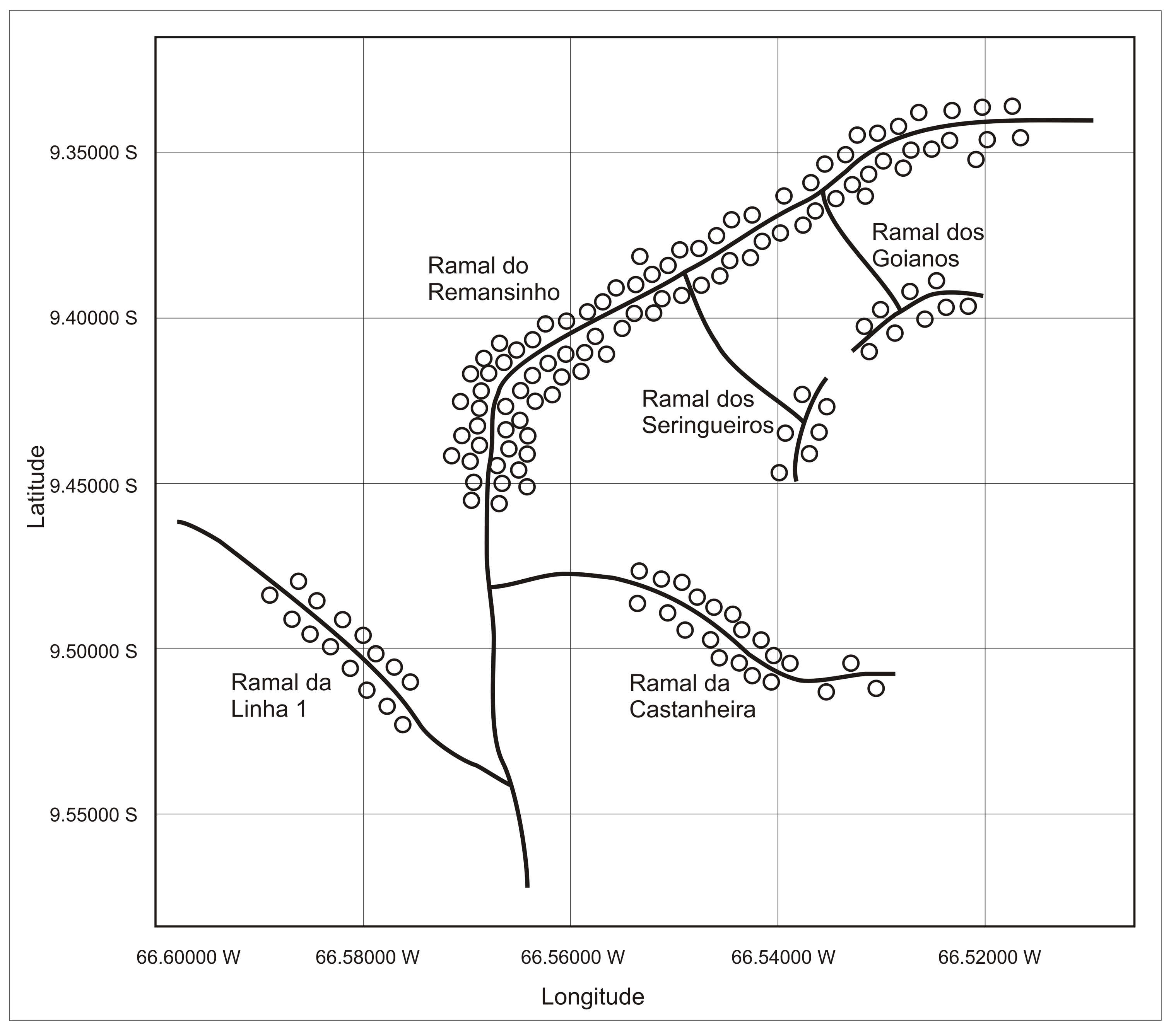

Supplement: Figure S1 — Map showing the five human settlements within Remansinho area. Open circles are approximate locations of the households with study subjects. (TIF) [file pntd.0003109.s001.tif]

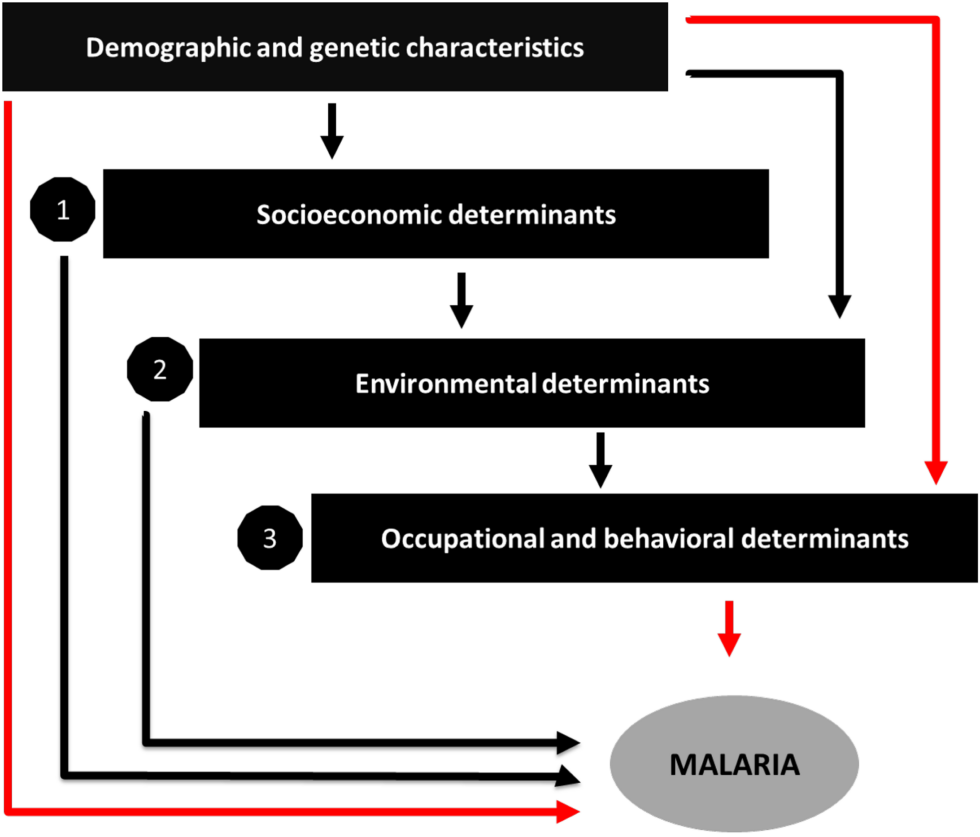

Supplement: Figure S2 — Conceptual hierarchical framework used to evaluate risk factors for P. vivax infection and clinical vivax malaria in mixed effects logistic regression models. (TIF) [file pntd.0003109.s002.tif]
