# Supplementary material for: Epidemiology of Disappearing Plasmodium vivax Malaria: A Case Study in Rural Amazonia
Source: PLoS Negl Trop Dis. 2014 Aug 28;8(8):e3109. doi: 10.1371/journal.pntd.0003109 (PMC4148206; doi:10.1371/journal.pntd.0003109)
Supplement: Methods S1 — Detailed description of the molecular methods used to detect malaria parasites and gametocytes, as well as of the hierarchical approach used to model risk factors for P. vivax infection and clinical vivax malaria. This supplement includes Figure S2. (DOCX) [file pntd.0003109.s004.docx]

**Methods S1**

#### Malaria diagnosis by quantitative PCR

#### DNA templates for PCR amplification were isolated on a QIAcube automated platform (Qiagen, Hilden, Germany), using QIAamp DNA blood kits (Qiagen), from either 200 μL of whole venous blood kept at -20°C or three 3-mm finger-prick bloodspots (corresponding to approximately 5 μL of whole blood), which were excised from FTA Micro Cards (Whatman, Clifton, NJ) kept at 4°C since blood collection. Final DNA elution volumes were 20 μL for bloodspots and 200 μL for whole blood. Each 20 µL reaction mixture contained 2 µL of sample DNA eluted by either method (corresponding to approximately 2 µL of venous blood or 0.5 µL of finger-prick blood), 10 µL of 2× Maxima SYBR Green qPCR master mixture (Fermentas, Burlington, Canada) and 0.5 μM of each primer. We used the genus-specific primer P1 (ACG ATC AGA TAC CGT CGT AAT CTT) combined with either of the species-specific primers, V1 (CAA TCT AAG AAT AAA CTC CGA AGA GAA A) or F2 (CAA TCT AAA AGT CAC CTC GAA AGA TG), for *P. vivax* and *P. falciparum,* respectively [22]. These primers allow the amplification of species-specific 100-base pair (bp) fragments from three (*P. vivax*) or five (*P. falciparum*) *18S rRNA* gene paralogs found in the assembled genomes of these species (Sal-I and 3D7 strains, respectively). Standard curves were prepared with serial tenfold dilutions of the target sequence, cloned into pGEM-T Easy vectors (Promega, Madison, WI), to allow for species-specific quantitation of parasite loads (number of parasites/μL of blood). For subjects co-infected with *P. vivax* and *P. falciparum,* parasite counts for each species were summed to calculate the total parasitemia. We used a Step One Plus Real-Time PCR System (Applied Biosystems, Foster City, CA) for PCR amplification with a template denaturation step at 95°C for 10 min, followed by 40 cycles of 15 sec at 95°C and 1 minute at 60°C, with fluorescence acquisition at the end of each extension step. Amplification was immediately followed by a melting program consisting of 15 sec at 95°C, 15 sec at 60°C, and a stepwise temperature increase of 0.2°C/sec until 95°C, with fluorescence acquisition at each temperature transition. Standard curves accompanying each assay indicated that the detection threshold of this diagnostic qPCR is approximately 2 parasites/µL of blood for *P. falciparum* and 3.3 parasites/µL of blood for *P. vivax*. No-template controls (containing all reagents for amplification except for the DNA template) were run for every qPCR microplate.

**Detection of *Plasmodium vivax* gametocyte-specific gene transcripts by quantitative reverse transcriptase PCR**

Venous blood samples collected from 55 *P. vivax* infections during cross-sectional surveys 4, 5, and 6 were cryopreserved at -70°C or in liquid nitrogen in our field laboratory and shipped to São Paulo in liquid nitrogen for RNA isolation. All samples yielded a positive qPCR for *P. vivax* only, being 27 missed by microscopy and 28 confirmed by microscopy. We processed 200-μl aliquots of these cryopreserved samples using the QIAmp Viral RNA Mini Kit (Qiagen) and eluted RNA was treated with RNAse-free DNAse (Fermentas) for removal of residual genomic DNA from templates for complementary DNA synthesis. For SYBR green qRT-PCR, we used the oligonucleotide primers (forward, AAC GAA GGG CTG GTG CAC CTT T; reverse, AGC AAC CTG CAC TTT GGA TTT CCG) designed by Bharti and colleagues [25] to amplify a 267-bp fragment of the *Pvs25* gene. This gene fragment was cloned into the pGEM-T Easy plasmid vectors (Promega) to prepare a standard curve used in each PCR microplate. Real-time PCR was carried out in triplicate, on a Mastercycler realplex S real-time thermal cycler (Eppendorf, Hamburg, Germany)*.* Each 15-µL reaction mixture contained 2 µL of sample cDNA, 7.5 µL of 2× Maxima SYBR Green qPCR master mixture (Fermentas) and 0.3 μM of each oligonucleotide primer. Amplification included a template denaturation step at 95° C (2 min) followed by 35 cycles of 30 sec at 95°C, 35 sec at 55°C and 2.5 min at 68°C, with fluorescence acquisition at the end of each extension step and a final extension step of 10 min at 68°C, with a final melting program consisting of 15 sec at 95°C, 15 sec at 60°C, and a stepwise temperature increase of 0.03°C/sec until 95°C. Fluorescence acquisition was done at each temperature transition. Quantitative results were expressed as numbers of *Pvs25* gene transcripts/µL of blood. The following negative controls were used: (a) to control for genomic DNA contamination, a RT-minus control (containing all reagents for reverse transcription except for the Maxima enzyme mix [Fermentas]) was run for each RNA sample, using the standard qRT-PCR protocol reagents; (b) to control for reagent contamination, no-template controls (containing all reagents for reverse transcription except for the RNA template) were run for every PCR microplate. As a control for cDNA template integrity, for each sample we run a qPCR targeting the *18S rRNA* gene of *P. vivax* (described above)*,* which is expressed by all parasite's blood stages. RNA isolation and cDNA synthesis were repeated whenever amplification of the *18S rRNA* control product failed. As a positive control for *pvs25* gene amplification, genomic *P. vivax* DNA templates were run for every PCR microplate.

**Hierarchical modeling of risk factors for infection and disease**

*Plasmodium vivax* infection and progression to clinical malaria disease is a complex process in which there seems to be interrelationships between its determinants. Constructing a model with a hierarchical structure is a way to incorporate interdependence between some of the covariates, regarded as plausible on biological, socio-economic or other grounds.

Figure S2 shows how we incorporated in a hierarchical structure the relationships between the risk factors for *P. vivax* infection and clinical vivax malaria when adjusting mixed effects logistic regression models. The effects of distal determinants, such as demographic, social and environmental factors on malaria risk are often not direct, but mediated by more proximate determinants, such as occupational and behavioral factors. Variables included in each level were: (a) demographic and genetic characteristics – Duffy blood group genotype (initially stratified as Duffy-positive or Duffy-negative), sex, age (stratified as < 15 years and ≥ 15 years), and years of residence in Amazonia (continuous variable); (b) socioeconomic factors – wealth index, stratified into quartiles; (c) environmental determinants – place of residence in Remansinho (categorized as Ramal do Remansinho, Ramal da Linha 1, Ramal da Castanheira, or Ramal dos Seringueiros and Ramal dos Goianos grouped together), presence of walls in the bedroom, presence of gaps in the walls, and whether bedroom windows were left open at night; and (d) occupational and behavioral determinants – main occupation (categorized as housekeeping, agriculture and forestry, or students/minors), time the individual goes to sleep, time the individual wakes up, bathing time and place, and practice of recreational fishing.

Variables within each level were introduced in the model in a stepwise approach and retained whenever they were associated with the outcome at a significance level of 20% or we considered it necessary. Variables retained within a given level were retained in subsequent models, which incorporated more proximate variables. The more distal level included the wealth index, the only socioeconomic variable, stratified into quartiles. The next , intermediate, level included several environmental variables, such as place of residence in Remansinho area (either [a] Ramal do Remansinho, [b] Ramal da Linha 1, [c] Ramal da Castanheira, or [d] Ramal dos Seringueiros and Ramal dos Goianos grouped together), presence of walls in the bedroom and presence of gaps in the walls, and whether bedroom windows were left open at night. The last, proximate, level comprised occupational and behavioral variables potentially associated with exposure to malaria, such as main occupation (classified as housekeeping, agriculture and forestry, or students/minors), sleeping habits (sleeping and waking-up time), bathing time and place, and recreational fishing.

**Figure S2 legend**. Conceptual hierarchical framework used to evaluate risk factors for *P. vivax* infection and clinical vivax malaria in mixed effects logistic regression models. Red arrows indicate that demographic and genetic characteristics of the study subjects may affect the outcome either directly or through more proximate determinants of infection, such as occupational and behavioral factors (see main text for details).
